# Assessing a decade of leukaemia-related premature mortality costs: impact on productivity loss in Spain

**DOI:** 10.1007/s10198-024-01727-6

**Published:** 2024-09-28

**Authors:** Josep Darbà, Meritxell Ascanio, Ainoa Agüera

**Affiliations:** 1 Department of Economics, Universitat de Barcelona, Diagonal 696, Barcelona, 08034 Spain; 2 BCN Health Economics & Outcomes Research S.L. Travessera de Gràcia, 62, Barcelona, 08006 Spain

**Keywords:** Leukaemia, Years of potential productive life lost, Productivity costs, Human capital approach.

## Abstract

**Introduction:**

Cancer mortality is one of the dominant causes of productivity loss; and within all cancer sites, blood cancer is the fourth most common cause of death in Spain. Thus, its impacts in work productivity are a major concern and represent a high social impact. The aim of this study was to evaluate the productivity losses resulting from of premature deaths due to leukaemia in Spain.

**Methods:**

The productivity costs stemming from premature mortality due to leukaemia were estimated using the human capital method. Information pertaining to mortality rates, typical incomes, and joblessness figures was gathered throughout a decade-long period spanning from 2012 to 2021.

**Results:**

Leukaemia caused 40% of haematological malignancies losses. It represented a 3.39% of all cancer-related deaths. In addition, it was responsible for 7,851 years of potential productive life lost (YPLPLL) in 2021, and productivity losses of €4,206.52 million over the 10-year period. All these numbers are relevant for Spain as will help on a more efficient distribution of resource.

**Conclusions:**

These productivity losses obtained, highlight the burden of leukaemia on the Spanish population, providing novel data on the number of deaths, trends and productivity losses for this type of cancer. This evaluation offers fresh insights that can aid policymakers in efficiently distributing resources, thereby lessening the economic burden it imposes on individuals of working age.

## Introduction

Cancer is a global health concern and cancer incidence, and mortality are rising quickly [1–4]. According to the World Health Organisation, it is the most important cause of death and morbidity in Europe (20%) after cardiovascular diseases. With more than 3.7 million new cases and 1.9 million deaths per year, cancer is the second leading cause of death and morbidity in Europe [5]. Premature mortality, defined as death before life expectancy, has an important effect on the economy. Consequently, if a person dies prematurely and was in paid work, this represents a loss of productivity for society, but also a loss of unpaid work, i.e. work that people do in their daily lives for which they do not receive any direct compensation, such as caring for a family member. Productivity losses are an important part of the disease’s economic burden, so it is important to estimate its impact.

The evaluation of productivity loss provides important data for informed resource allocation. Different approaches may be used to evaluate the productivity loss [6]. The human capital approach is the most common method, if individuals have an implicit productivity up to their retirement age that is diminished due to illness or death, so that losses can be quantified [7]. A second approach is the friction cost, which focuses on the losses relative to the time lost in trying to replace a worker [8]. The aim of this method is to provide a more realistic estimate; however, a standard measure of replacement time is required [9]. Lastly, other methods focus on other variables, for example, the willingness-to-pay method, which assesses intangible costs such as suffering and discomfort by assigning them a monetary value [10].

Cancer-related productivity losses provide an important alternative indicator of the burden of cancer in society and have the potential to support policy decisions. According to a study published in 2020, the productivity loss from premature mortality due to cancer accounted for €50 billion in Europe [11]. In Spain, the total value of productivity lost due to premature cancer mortality in 2018 was estimated as €7550 million [12]. Information about leukaemia productivity loss is missing and just some data about acute myeloid leukaemia (AML) productivity impact has been published [13]. The study by Parker et al. shows that the productivity loss attributable to AML in the Australian population over 10 years was 7,600 life years lost, representing US$971 million. The average age of diagnosis of leukaemia is over 65 years, and this may be the reason why the productivity losses of this disease have not yet been assessed [14–16].

Estimates of the economic impact of cancer-related productivity loss have been made in several countries [17, 18]. For example, in Spain, it was estimated that temporary incapacity due to cancer caused an economic loss of 248.6 million euros in 2005 [19]. In addition, premature mortality from cancer is estimated to have caused losses of 2.5 billion euros in 2009 [20]. These significant costs highlight the burden of cancer on the economy and emphasise the potential economic benefits that could be realised through the implementation of well-informed policies aimed at reducing the incidence of the costliest cancer types.

In the recent years some studies aimed to assess the direct costs borne by healthcare systems in Europe resulting from premature deaths due to leukaemia. Indirect costs (i.e., productivity loss) should also be included in those studies to have a complete picture of the leukaemia burden. The concrete aim of this study was to assess the productivity losses resulting from premature deaths due to leukaemia in Spain.

## Materials and methods

### Model and methodology

The costs of premature leukaemia mortality were calculated using the human capital (HC) approach, which estimates the income and productivity of an individual that is avoided when a premature death occurs [7]. It is the most widely used method for assessing productivity losses (paid and unpaid) and shows the negative effects on an individual’s health may lead to adverse effects on his or her productivity at work and at home and may also lead to a loss of free time. It considers that the loss covers the period between the age at which an individual dies prematurely and the assumed age of retirement.

Using the HC theory, we applied a simulation model to estimate the present and future productivity loss due to premature deaths caused by leukaemia, considering the age at death of each individual, as well as the employment rate and wage by sex and age. We restrict our estimation to labour productivity losses, and do not consider productivity losses due to unpaid work or leisure time in our estimation. For each age group, the number of deaths was multiplied by the average remaining life expectancy. Therefore, the number of years of potential life lost (YPLL) due to the premature deaths of *n* individuals was calculated as follows, where L is the mean remaining life expectancy for each age and sex:

$$\:\text{Y}\text{P}\text{L}\text{L}=\sum\:_{i=1}^{n}{L}_{i}$$

Then, the number of years of potential labour productive life lost (YPLPLL) was calculated. All deaths before the age of 65, considered as the retirement age, were considered. The YPLPLL was calculated by multiplying the number of deaths for a specific age group by the expected years of remaining productive life for each age group. The number of YPLPLL was calculated as follows, where *Wu* is the maximum limit of the working age (65 years) and *Wl* is the age at death:

$$\:\text{Y}\text{P}\text{L}\text{P}\text{L}\text{L}=\sum\:_{i=1}^{n}{Wu}_{i}-{Wl}_{i}$$

Lastly, the calculated YPLPLL was multiplied by the age- and sex-specific wages, adjusted by the employment rate, between the age of death and the age of retirement. Thus, labour productivity losses (LPL) can be estimated as follows, where S is the age- and sex-adjusted wage, and e is the age- and sex-adjusted employment rate:

$$\:\text{L}\text{P}\text{L}=\sum\:_{i=1}^{n}{\text{Y}\text{P}\text{L}\text{P}\text{L}\text{L}}_{i}*{S}_{i}*{e}_{i}$$

### Datasets

The study period was set at 10 years, between 2012 and 2021, to cover the most recent data available from the INE registries. To obtain the data on leukaemia related fatalities, we have used the INE data from the Death Registry [21]. This data included details on the age and gender of the deceased individuals. To gather insights into the employment rate, data from the Labour Force Survey, conducted by INE as well, was used [22]. Lastly, data on remuneration, encompassing both monetary compensation and non-monetary benefits, for work performed was obtained from the Spanish Structural Wage Survey, performed by INE [23].

### Output

The YPLPLL were calculated by using the developed model where the current and anticipated labour productivity decline resulting from premature deaths, accounting for factors such as the age at which individuals pass away, employment rates, and gender- and age-specific wages. Our estimation is focused exclusively on labour productivity losses and does not encompass unremunerated work or leisure time-related productivity losses in our calculation [24]. The retirement age was set at 65 years, according to the Spanish legislation [25].

To estimate the costs of premature mortality, sex- and age-specific annual salaries were applied from the age of death to the age of retirement [26].

### Scenario analyses

An annual discount rate of 3% was used in the baseline case for future income values and a scenario analysis was run considering two alternative discount rates, 0% and 6%. An additional analysis was carried out considering the retirement age at 67, in line with new legislation in Spain that has delayed the retirement age in recent years [25].

## Results

In total, 33,751 people died from leukaemia in Spain between 2012 and 2021, 5,856 of them of working age (Table 1). Leukaemia deaths accounted for 3.39% of all cancer-related deaths. The year 2012 showed the highest number of YPLPLL in males with 5,643. The same year also showed the highest number in females with 4,285. In the total study period, the average annual number of YPLPLL was 8,562 (Table 1).

**Fig. 1:**
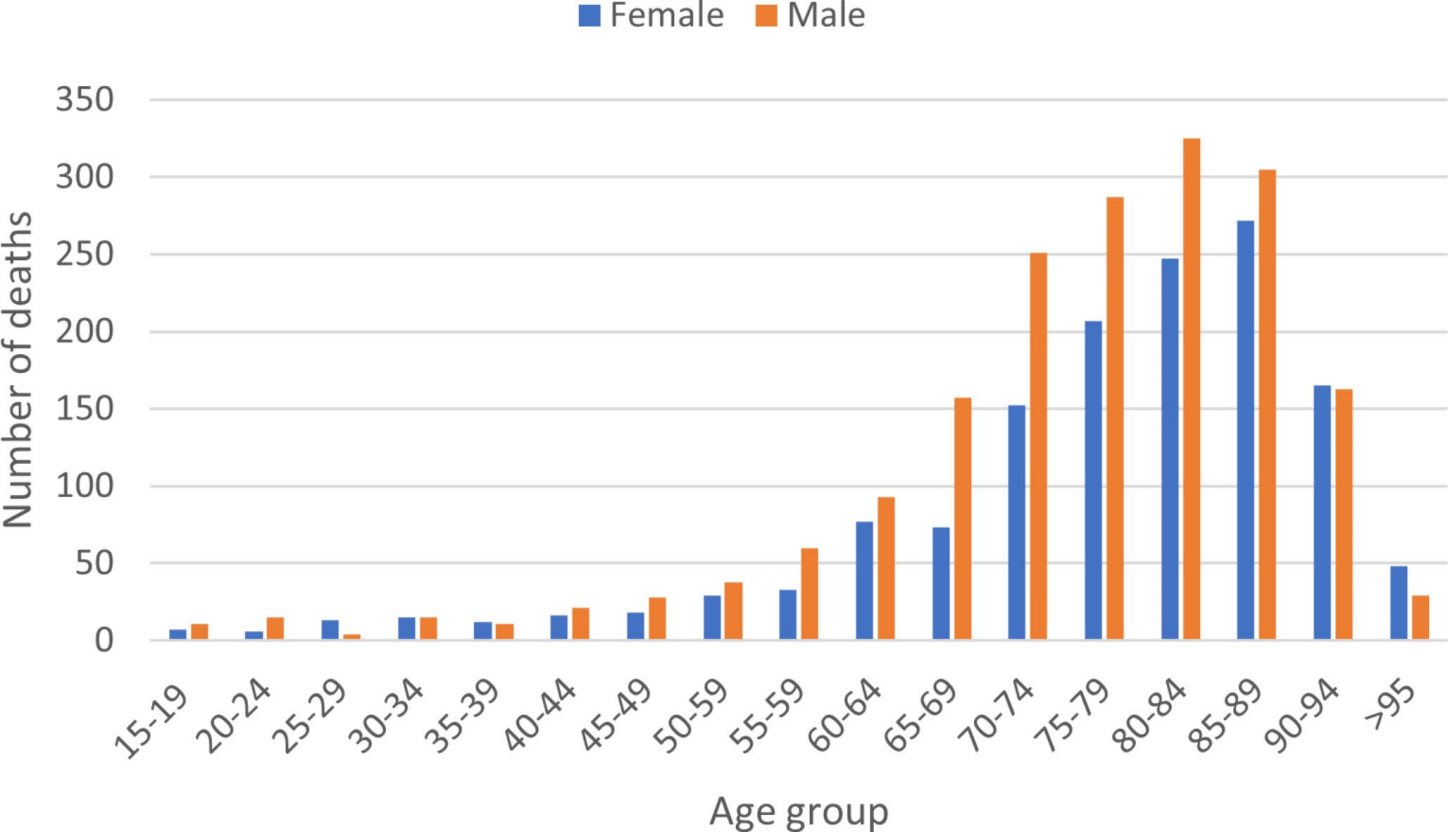
Number of deaths per each age group studied in 2021

As illustrated in Fig. 1, a higher incidence of leukaemia deaths is observed among males. Additionally, it is worth noting that age groups over 65 years of age exhibit a notable concentration of patients, suggesting that a substantial portion of individuals are diagnosed with leukaemia after reaching the retirement age.

**Table 1:** Measures of deaths, portion of cancer-related deaths attributable to leukaemia and years potential productive life lost (YPLPLL) due to leukaemia

Year	2012	2013	2014	2015	2016	2017	2018	2019	2020	2021
Number of deaths										
*males*	1,925	1,912	1,917	1,969	1,916	1,926	1,895	1,921	1,876	1,813
*females*	1,547	1,499	1,416	1,563	1,459	1,484	1,453	1,431	1,439	1,390
% Deaths at working age										
*males*	20.8	20.1	18.2	17.8	19.8	18.5	18.8	15.6	17.4	16.3
*females*	18.1	15.7	16.6	16.8	15.2	15.9	14.2	15.4	16.1	16.3
% Leukaemia vs. all tumors										
*males*	2.7	2.7	2.7	2.7	2.7	2.7	2.7	2.7	2.7	2.7
*females*	4.3	4.2	4.2	4.2	4.1	4.1	4.1	4.0	4.0	4.0
YPLPLL										
*male*	5,643	5,470	5,452	5,363	5,565	5,336	4,801	3,996	4,801	4,368
*female*	4,285	3,348	3,520	4,099	3,566	3,103	2,668	3,088	3,756	3,483

YPLPLL: years of potential productive life lost

The costs of premature mortality were estimated, predicting productivity losses up to the retirement years and adjusting all measures to age- and gender-specific annual wages. The analysis provided three estimates, a baseline and the results of the sensitivity analysis. The cumulative productivity losses from 2012 to 2021 caused by leukaemia were 1.4 billion euros. The most recent available data shows that costs due to productivity losses caused by leukaemia were142 million euros in 2021 (Table 2).

**Table 2:** Productivity losses (in millions) due to leukaemia (annual costs discount rates)

Year	Premature mortality costs (baseline)	Premature mortality costs (0%)	Premature mortality costs (6%)
2012	154	158	150
2013	134	137	130
2014	132	135	128
2015	144	148	140
2016	145	149	141
2017	143	147	139
2018	133	136	121
2019	124	128	148
2020	152	156	138
2021	142	145	136
Total	1,401	1,440	1,371

The scenario analyses considering the different annual discounts rates ranged between 1.37 and 1.44 billion euros (Table 2). When assessing a different retirement age, set at 67 years old, the results accounted for a total of 2.1 million euros from 2012 to 2021 (Table 3).

**Table 3:** Productivity losses (in millions) due to leukaemia (retirement age)

Year	Premature mortality retirement age (baseline)	Premature mortality Retirement age (67 years old)
2012	154	226
2013	134	201
2014	132	196
2015	144	212
2016	145	222
2017	143	222
2018	133	210
2019	124	199
2020	152	232
2021	142	217
Total	1,401	2,137

**Fig. 2:**
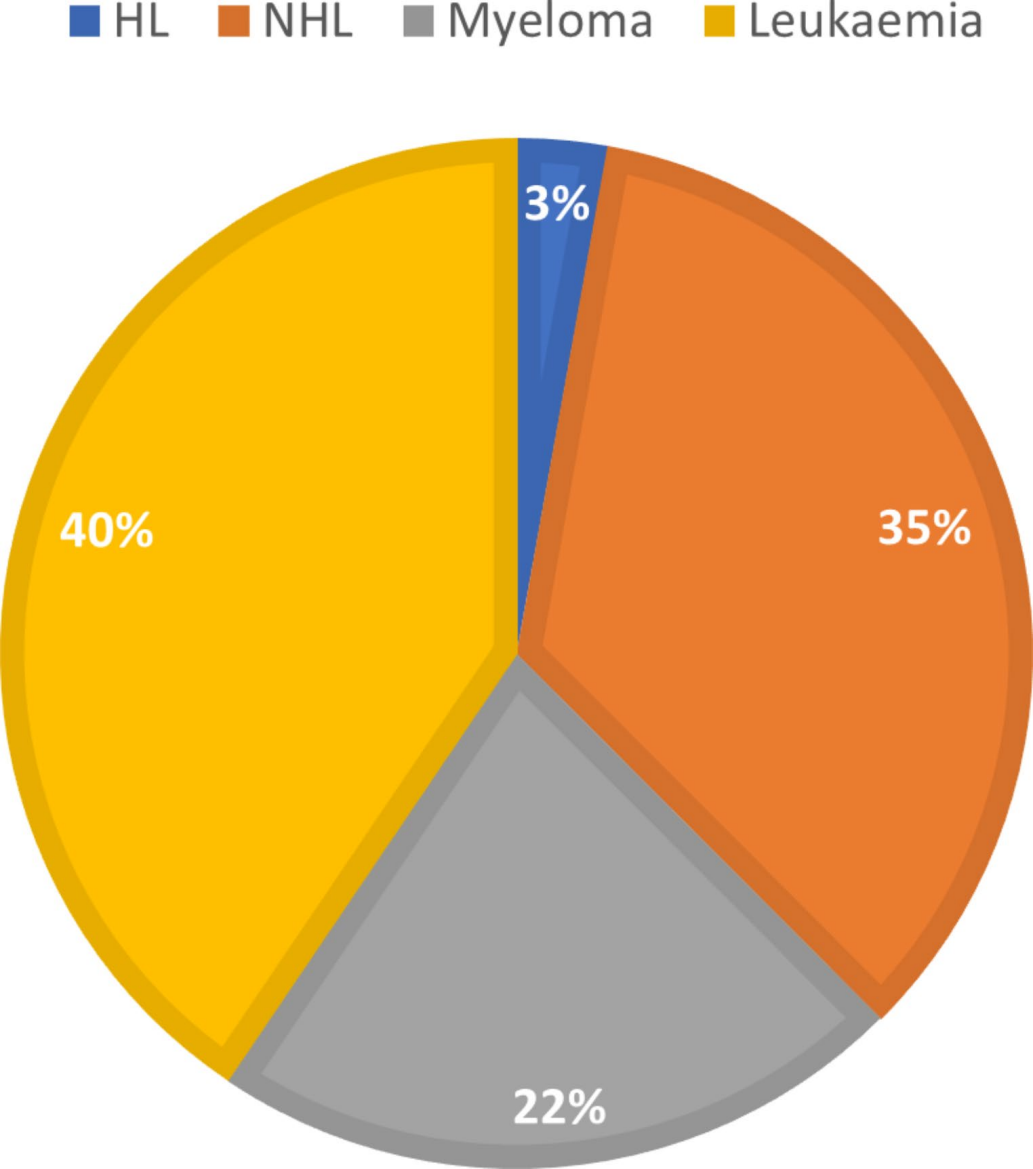
Portion of haematological malignancies losses HL: Hodgkin’s lymphoma; NHL: non-Hodgkin’s lymphoma

Leukaemia constituted a substantial portion, amounting to 40% of the overall losses attributed to haematological malignancies, when considering other conditions such as myeloma, Hodgkin’s lymphoma (HL), and non-Hodgkin’s lymphoma (NHL) (Fig. 2).

## Discussion

This study showed that a total of 33,751 people died from leukaemia in Spain between 2012 and 2021, representing an annual average of 8,562 YPLPLL were lost in the study period. Leukaemia deaths accounted for 3.39% of all cancer-related deaths. Additionally, the costs of lost productivity due to premature mortality from leukaemia are estimated at 1.4 billion euros during the study period. When considering all the haematological malignancies, leukaemia constituted 40% of the overall losses.

As far as the authors are aware, this is the first study to estimate productivity costs due to premature leukaemia deaths in Spain, which will be a major breakthrough in the calculation of the economic burden of leukaemia.

Our study found that in 2021, 16.33% of working-age males died of leukaemia, while 16.26% of females died of leukaemia. Although the incidence of death is similar, there is a gender bias in the incidence of leukaemia, as men are more frequently diagnosed with leukaemia [26]. Particularly, some types of leukaemia such as acute myeloid leukaemia are known to be more common in men, although the reasons for this difference are still not known [27, 28]. There has been a slight decrease in the number of deaths over the decade, which may be related to new technologies to detect early cases. Incidence increases with age, with the most affected age group being between 80 and 84 years of age, in both sexes.

It is reasonable to assume that several constraints may have influenced the results of this study. The human capital approach is the most widely used method for estimating the indirect costs of illness through productivity losses, although it has several limitations. Some previous studies have assessed and compared results using other approaches as the friction costs [29]. Rissanen et al. showed that there are high differences between the estimation with both methods, with productivity costs of stroke patients of €166,050 when assessed using the HC method and €7,020 when using the friction cost method [29]. One limitation of our analysis that we acknowledge is the constrained scope of our findings, as the data availability does not permit us to extrapolate our results to encompass labour productivity losses arising from absenteeism or presenteeism. Bradley et al. demonstrated that cancer surviving patients have the highest rate of absenteeism among chronically ill patients [30]. Additionally, our analysis does not extend to the realm of productivity losses stemming from unpaid work or other time-related losses, such as leisure. The assumption that future benefits are converted into productivity has been questioned, along with the deliberation that lost productivity is not incorporated by another individual [31]. Ultimately, treatments have progressed and become more effective over time. Additionally, there is uncertainty regarding the specific treatments administered to the patients and the effects that may have on the productivity of these patients.

In addition, although some studies assessed the direct costs of leukaemia, indirect costs (i.e., productivity loss) should also be included in those studies to have a complete picture of the leukaemia burden [32]. These costs are usually not included because are difficult to estimate, and there is no consensus on a better estimation approach [33]. Although some evaluation agencies recommend the inclusion of social costs, there is a wide variation in the specific type of costs recommended for inclusion [34].

Finally, early detection of any type of cancer often allows for more treatment options. Detecting cancer early can significantly reduce the mortality associated with the disease. In addition, treatment of cancer in early stage is generally more effective, less complex and less expensive. Not only does early detection save lives, but it also allows people to continue working and supporting their families while accessing timely and effective treatment. In the case some new screening options for leukaemia detection come to the market, the findings from this study may help decision-makers to allocate resource and prioritise early detection programmes.

## Conclusion

Overall, productivity costs due to leukaemia mortality have been assessed. Leukaemia accounted for 40% of all losses due to haematological malignancies, with 7,851 YPLPLL expected and accounting for €141.56 million in lost productivity in 2021 in Spain. These data highlight the burden of leukaemia on the Spanish population, providing novel data on the number of deaths, trends and productivity losses symbolised by this type of cancer. This can help decision-makers in the allocation of resources, prioritising early detection screening programmes that are likely to produce substantial decreases in mortality and productivity losses.

## Data Availability

The data that support the findings of this study are available from the Spanish national statistics institute at http://www.ine.es.
